# Applying semantic web technologies for phenome-wide scan using an electronic health record linked Biobank

**DOI:** 10.1186/2041-1480-3-10

**Published:** 2012-12-17

**Authors:** Jyotishman Pathak, Richard C Kiefer, Suzette J Bielinski, Christopher G Chute

**Affiliations:** 1 Division of Biomedical Statistics and Informatics, Department of Health Sciences Research, Mayo Clinic, Rochester, MN, USA; 2 Department of Information Technology, Mayo Clinic, Rochester, MN, USA; 3 Division of Epidemiology, Department of Health Sciences Research, Mayo Clinic, Rochester, MN, USA

## Abstract

**Background:**

The ability to conduct genome-wide association studies (GWAS) has enabled new exploration of how genetic variations contribute to health and disease etiology. However, historically GWAS have been limited by inadequate sample size due to associated costs for genotyping and phenotyping of study subjects. This has prompted several academic medical centers to form “biobanks” where biospecimens linked to personal health information, typically in electronic health records (EHRs), are collected and stored on a large number of subjects. This provides tremendous opportunities to discover novel genotype-phenotype associations and foster hypotheses generation.

**Results:**

In this work, we study how emerging Semantic Web technologies can be applied in conjunction with clinical and genotype data stored at the Mayo Clinic Biobank to mine the phenotype data for genetic associations. In particular, we demonstrate the role of using Resource Description Framework (RDF) for representing EHR diagnoses and procedure data, and enable federated querying via standardized Web protocols to identify subjects genotyped for Type 2 Diabetes and Hypothyroidism to discover gene-disease associations. Our study highlights the potential of Web-scale data federation techniques to execute complex queries.

**Conclusions:**

This study demonstrates how Semantic Web technologies can be applied in conjunction with clinical data stored in EHRs to accurately identify subjects with specific diseases and phenotypes, and identify genotype-phenotype associations.

## Introduction

In the past decade, there has been a plethora of discoveries in genomic sciences involving complex, non-Mendelian diseases that relate single-nucleotide polymorphisms (SNPs) to clinical conditions and measurable traits. This has become feasible due to the advances in high-throughput genotyping technologies and genome-wide association studies (GWAS) that allow studying the entire human genome in thousands of unrelated individuals regarding genetic associations with different diseases. However, unlike Mendelian traits, effect sizes of genetic variants associated with common diseases are relatively small, and thus large sample sizes are required for discovery.

To address this research need, several academic medical centers are forming biorepositories or biobanks that collect and store individual biospecimens from which DNA for conducting genetic research can be extracted. Additionally, these biobanks are often linked to electronic health records (EHRs) that support retrieval and querying for vast amounts of phenotype data [1,2]. The Electronic Medical Records and Genomics (eMERGE [3]) consortium—a network of ten academic medical centers, of which Mayo Clinic is a member—has demonstrated the applicability of “EHR-derived phenotyping algorithms” for cohort identification to conduct GWAS for several diseases, including peripheral arterial disease [4], red blood cells [5] and atrioventricular conduction [6]. A common thread across the library of algorithms [7] is access to different types and modalities of clinical data for algorithm execution, which includes billing and diagnoses information, laboratory measurements, patient procedure encounters, medication and prescription management data, and co-morbidities (e.g., smoking history, socio-economic status). While on one hand these approaches with EHR-linked biorepositories have successfully facilitated GWAS, such studies typically focus on a narrow phenotypic domain, such as presence or absence of a given disease and ignore the potential power that can be gained through intermediate and sub-phenotypes, as well as considering pleiotropic associations. Furthermore, most existing GWAS results are based on populations with European descent, thereby limiting the understanding of genetic contribution to diseases and traits for other racial and ethnic populations. To this end, there has been an emerging interest in mining the human phenome via a “reverse GWAS” or a PheWAS (Phenome Wide Association Scan)—for a given genotype, the goal is to identify the set of associated clinical phenotypes. By using clinical data from EHRs, a PheWAS allows systematic study of associations between a number of common genetic variations and variety of large number of clinical phenotypes. Recent studies by Denny et al [8]. and Pendergrass et al [9]. demonstrated the potential for PheWAS to replicate previously published genotype-phenotype associations, as well as, identify novel associations using patient EHR data. However, to extract phenotype data from EHRs, one is posed with the challenge of representing and integrating data in a form that would allow federated querying, reasoning, and efficient information retrieval across multiple sources of clinical data and information.

The work proposed in this study is an attempt to address this challenge by exploring and experimenting with Semantic Web technologies for enabling a PheWAS. A key aspect of Semantic Web is a rigorous mechanism for defining and linking heterogeneous data using Web protocols and a simple data model called Resource Description Framework (RDF). By representing data as labeled graphs, RDF provides a powerful framework for expressing and integrating any type of data. As of March 2012, under the auspices of an initiative called the Linked Open Data (LOD [10]), more than 250 public datasets from multiple domains (e.g., gene and disease relationships, drugs and side effects) are available in RDF, and have been integrated by specifying approximately 350 million links between the RDF graphs. Not only do such efforts provides tremendous opportunities to devise novel approaches for combining private, and institution-specific EHR data with public knowledgebases for phenotyping, but they also present several challenges in representing EHR data using RDF, creating linkages between multiple disparate RDF graphs, and developing mechanisms for executing federated queries analy-zing information spanning genes, proteins, pathways, diseases, drugs, and adverse events.

In this paper, we describe our efforts in representing real patient data, both clinical and genomic, from Mayo Clinic’s EHR systems [11] and the biobank, respectively as RDF graphs. In particular, we leverage open-source tooling and infrastructure developed within the Semantic Web community to extract phenotype and genotype information on subjects with Type 2 Diabetes Mellitus (T2DM) or Hypothyroidism, and conduct a phenome-wide scan to discover new genetic associations, as well as, replicate existing ones. As a proof of concept, we present our results on eight SNPs associated with T2DM and Hypothyroidism within an EHR population at the Mayo Clinic biobank. Our approach highlights the potential of using Semantic Web technologies for exploring a variety and large range of clinical phenotypes derived from EHRs for genomics research in a very high-throughput manner.

## Background

### Mayo Clinic Biobank and the genome consortia

The Mayo Clinic biobank is an institutional resource for biological specimens, patient provided risk factor data, and clinical data on patients recruited to the biobank. Operational since 2009, the biobank has enrolled more than 22,000 subjects in an effort to support a wide array of health-related research studies throughout the institution. Study participants provide a blood sample for DNA and serum/plasma research, complete a health risk questionnaire, allow access to medical records, and consent to prospective follow-up for health outcomes. Within this biobank, Mayo Genome Consortia (MayoGC [12]) is a large cohort of Mayo Clinic patients with clinical data (linked via their EHRs) and genotype data. Formed as a voluntary collaboration of investigators across disciplines at Mayo Clinic, eligible participants in MayoGC include those who gave general research (i.e., not disease-specific) consent to share high-throughput genotyping data with other investigators. The MayoGC cohort is being built in 2 phases. Phase I, which has been completed, includes participants from 3 studies funded by the U.S. National Institutes of Health (NIH) which sought to identify genetic determinants of peripheral arterial disease, venous thromboembolism, and pancreatic cancer, respectively, with a combined total sample size of 6,307 unique participants (Table 1). Phase 2 is currently underway with the goal of expanding MayoGC by recruiting eligible patients from several additional studies funded by the NIH and other governmental and non-profit agencies at Mayo Clinic. For this study, we extracted clinical and genotype data on all 6,307 subjects from Phase I (Table 1).

**Table 1:** MayoGC Phase I studies^a,b^**(used with permission from Bielinski et al [**[12]]**)**

**Characteristics**	**eMERGE Network (PAD)**[2]		**GENEVA (VTE)**[6]		**PANC**[7,8]
	Cases (n = 1612)	Controls (n = 1585)	Cases (n = 1233)	Controls (n = 1264)	Controls (n = 613)
Age (y), mean ± SD	66.0 ± 10.7	61.0 ± 7.4	55.0 ± 16.2	56.0 ± 15.8	66.0 ± 10.0
Female (%)	36	40	50	52	45
Medical record length (y)					
Mean ± SD	23.4 ± 20.0	26.1 ± 20.3	13.7 ± 16.3	21.1 ± 15.4	30.2 ± 16.5
Median ± (range)	18.7 (1.0–78.6)	23.0 (1.0–79.2)	6.3 (1.0–71.8)	17.8 (1.0–70.2)	29.8 (1.0–75.0)
White (%)	94	94	96	99	100
Geographic location, No. (%)^c^					
Olmsted Country	328(20)	590(37)	7(1)	10(1)	64(10)
Southeast Minnesota	191(12)	62(4)	205(17)	378(30)	107(17)
Greater Minnesota	393(24)	343(22)	314(25)	371(25)	135(22)
Iowa	212(13)	97(6)	176(14)	191(15)	65(11)
South and North Dakota	50(3)	31(2)	79(6)	71(6)	19(3)
Wisconsin	128(8)	68(4)	121(10)	138(11)	32(5)
Other states or international	309(19)	394(25)	330(27)	159(13)	191(31)

^a^eMERGE=Electronic Medical Records and Genomics; GENEVA=Gene Environment Association Studies; MayoGC=Mayo Genome Consortia; PAD=peripheral arterial disease; PANC=Mayo Clinic Molecular Epidemiology of Pancreatic Cancer Study; VTE=venous thromboembolism.

^b^Percentages may not total 100% because of rounding.

^c^Southeast Minnesota includes 7 counties in the southeast corner of Minnesota: Dodge, Goodhoue, Wabasha, Winona, Houston, Fillmore, and Mower, Olmsted County, Minnesota, is a mutually exclusive category.

### Genetics of type 2 diabetes mellitus

The prevalence of T2DM has been increasing rapidly in recent years with an estimated 438 million adults suffering from diabetes by the year 2030 [13]. While there are numerous non-genetic factors that contribute to the development of diabetes prevalence, recent studies indicate the importance of genetic findings for the pathophysiology, prediction, and treatment of T2DM [14]. Furthermore, association studies focusing on quantitative traits such as fasting glucose, fasting insulin, and glycated hemoglobin A1C (HbA1c) have shed further light on the genetic susceptibility of T2DM. To date, at least 36 gene loci have been identified that contribute to the genetic risk of T2DM, although this number is expected to increase in the future with larger cohorts being assembled. In particular, current estimates indicate that the gene loci that are associated with T2DM, explain only approximately 10% of the disease heritability. This raises the challenge for finding the remaining heritability as well as identification of additional diabetes-related gene loci that can be expected to lead to creation of clinically relevant disease prediction models. While a detailed discussion on genetics of T2DM is beyond the scope of this paper (interested readers can refer to Herder et al [14].), Table 2 below lists some of the gene loci and SNPs that are associated with T2DM or related traits.

**Table 2:** Examples of gene loci associated with T2DM, Hypothyroidism and related traits

**Gene locus**	**Full gene name**	**SNP**	**Associated phenotype**	**Odds ratio (95% CI)**	**p-value**	**Reference**
PPARG	Peroxisome proliferator-activated receptor gamma	rs1801282	T2DM	1.14 (1.08-1.20)	1.7 × 10^-6^	Scott et al [44].
KCNJ11	Potassium inwardly rectifying channel, subfamily J, member 11	rs5219	T2DM	1.14 (1.10-1.19)	6.7 × 10^-11^	Scott et al [44].
TCF7L2	Transcription factor 7-like 2	rs7903146	T2DM, glucose, HbA1c	1.37 (1.31-1.43)	1.0 × 10^-8^	Sladek et al [45].
		rs12255372				
SLC30A8	Solute carrier family 30 [zinc transporter], member 30	rs13266634	T2DM, HbA1c	1.12 (1.07-1.16)	5.3 × 10^-8^	Zeggini et al [46].
FTO	Fat mass and obesity associated	rs8050136	T2DM, BMI	1.17 (1.12-1.22)	1.3 × 10^-12^	Scott et al [44].
FOXE1	Forkhead box protein E1	rs965513	Thyroid cancer, TSH levels	1.75 (1.49-2.01)	1.7 × 10^-27^	Gudmundsson et al [18].
FOXE1	Forkhead box protein E1	rs7850258	Hypothyroidism	0.74 (0.67-0.82)	3.96 × 10^-9^	Denny et al [47].
PTPN22	Protein tyrosine phosphatase, non-receptor type 22	rs2476601	Hashimoto’s thyroiditis	1.77 (1.31-2.40)	4.6 × 10^-13^	Criswell et al [20].
VAV3	Guanine nucleotide exchange factor	rs4915077	Hypothyroidism	1.397 (1.27-1.54)	8.3 × 10^-11^	Eriksson et al [48].

### Genetics of hypothyroidism

Hypothyroidism is characterized by deficiencies of thyroid hormones T3 (triiodothyronine) and T4 (thyroxine) that are responsible for regulation of metabolic activities as well as growth and development. Primary hypothyroidism is the most common thyroid disorder affecting 1%--5% of the population [15], and up to 12% of the elderly express subclinical phenotypes of hypothyroidism [16]. Often marked by high thyroid-stimulating hormone (TSH), several GWA studies have found novel loci associations with TSH levels [17,18]. In a more recent thyroid cancer GWAS, Gudmundsson et al [19]. discovered associations between two SNPs and TSH levels near the genes *FOXE1* (forkhead box E1; also known as *TTF-2* thyroid transcription factor 2) and *NKX2-1* (NK2 homeobox 1; also known as *TTF-1* thyroid transcription factor 1). The predominant cause of hypothyroidism in the United States is an autoimmune disorder called—Hashimoto thyroiditis—where several candidate-gene analysis and linkage studies suggest that loci contributing to the pathogenesis of hypothyroidism include *CTLA4* (Cytotoxic T-Lymphocyte Antigen 4), *PTPN22* (Protein tyrosine phosphatase, non-receptor type 22) and *TG* (thyroglobulin) genes [20-22]. Table 2 provides additional information about gene loci and SNPs that are associated with hypothyroidism and related traits.

### Semantic web and related technologies for clinical and translational research

A key benefit of using Semantic Web technologies is its simple data model—RDF—that provides a rigorous mechanism of defining and linking data using Web protocols in a way, such that, the data can be used by machines not just for display, but also for automation, integration, and reuse across various applications. Furthermore, the availability of standard languages such as RDFS [23], OWL [24], and SPARQL [25] for creating ontologies as well as modeling and querying data, provides a very powerful framework for heterogeneous data integration. While most clinical and research data is typically stored using relational databases (e.g., Oracle, MySQL) and queried using Structured Query Language (SQL), such technologies have several inherent limitations compared to RDF: (i) First, when database schemas are changed in a relational model, the whole repository, table structure, index keys etc. have to be reorganized—a task that can be quite complex and time-consuming. RDF, on the other hand, does not distinguish between schema (i.e., ontology classes and properties) and data (i.e., instances of the ontology classes) changes—both are merely addition or deletion of RDF triples, making such a model very nimble and flexible for updates. (ii) Second, RDF resources are identified by (globally) unique IRI’s (international resource identifiers), thereby allowing anyone to add additional information about the resource. For example, via RDF links, it is possible to create references between two different RDF graphs, even in completely different namespaces, enabling much easier data linkage and integration. This is rather difficult to achieve in the classical relational database paradigm. (iii) Third, a relational data model lacks any inherent notion of a hierarchy. For instance, simply because a particular drug is an angiotensin receptor blocker (ARB), a typical SQL query engine (without any ad-hoc workarounds) cannot reason that it belongs to a class of anti-hypertensive drugs. Such queries are natively supported in RDFS and OWL. (iv) Finally, due to the lack of a formal temporal model for representing relational data, SQL provides minimal support for temporal queries natively. Such extensions are already in place for SPARQL [26].

In summary, Semantic Web and its enabling technologies such as RDF, provide a more robust, flexible, yet scalable model for integrating and querying data, thereby warranting investigation as to how such technologies can be applied in a clinical and translational research environment. However, while on one hand, such a huge integrated-network dataset provides exciting opportunities to execute expressive federated queries and integrating and analyzing information spanning genes, proteins, pathways, diseases, drugs, and adverse events, several questions remain unanswered about its applicability to high-throughput phenotyping of patient data in EHR systems.

## Methods

### System architecture: representing patient records and MayoGC genotype data as RDF graphs

**Figure 1:**
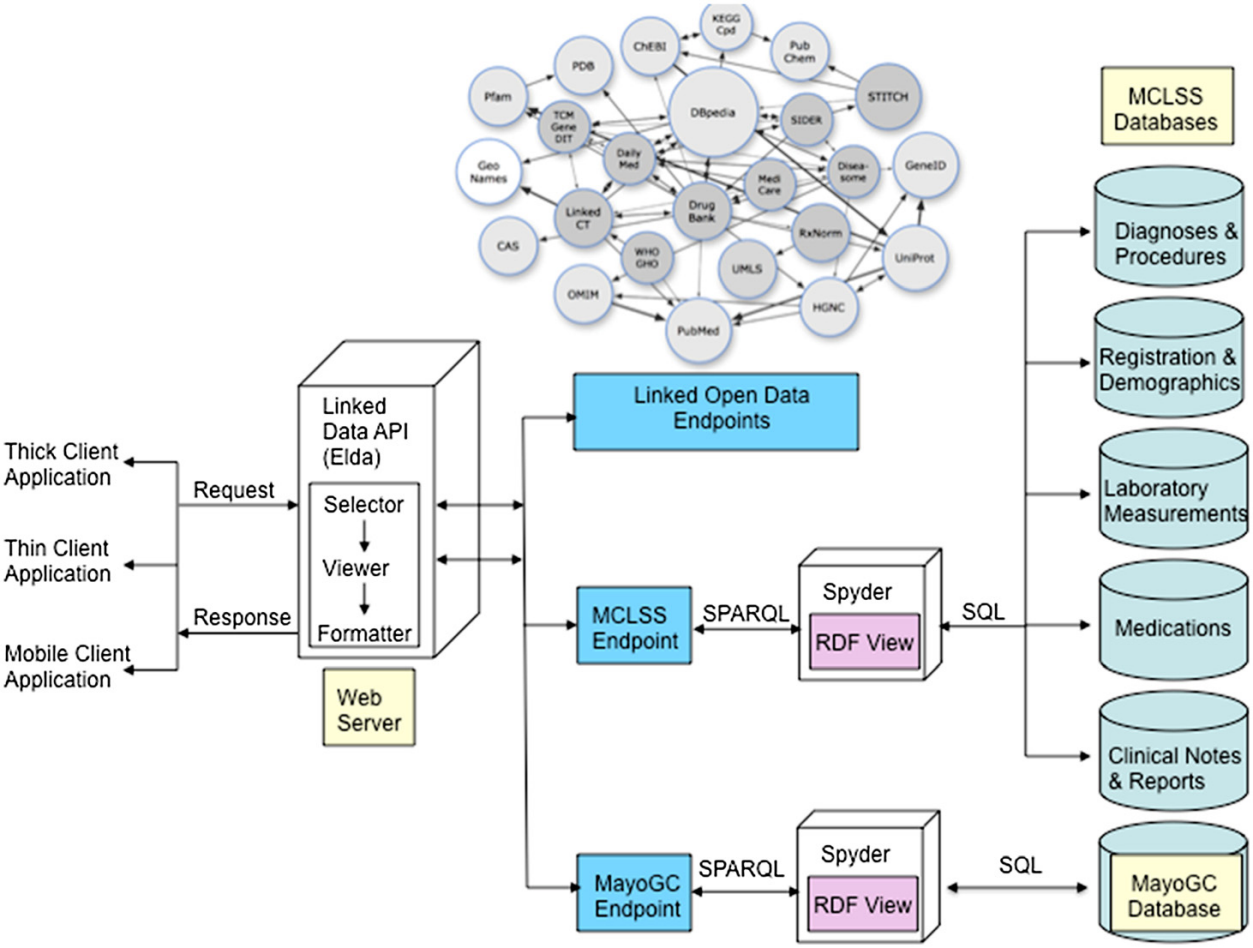
**System architecture for representing patient electronic health records and MayoGC data using RDF**.

Figure 1 shows our proposed architecture for representing patient health records and genotype data from MayoGC using RDF, linked data and related technologies. It comprises of three main components: (1) data access and storage, (2) RDF virtualization and ontology mapping, and (3) SPARQL-based querying interface. Here we provide a brief overview of these components, and more details were described in our prior work [27]. Where the prior work focused utilizing Semantic technologies to retrieve data from multiple tables within the same database, our current research expands that focus to retrieving data from multiple remote databases in order to add breadth and depth to the resultsets.

#### Data access and storage

This component comprises the patient demographics, diagnoses, procedures, laboratory results, and free-text clinical and pathology notes generated during a clinical encounter as well as SNP genotype data for all the 6,307 subjects from MayoGC (Table 1). For accessing the phenotype data, we leverage the Mayo Clinic Life Sciences System (MCLSS [28]) which is a rich clinical data repository maintained by the Enterprise Data Warehousing Section of the Department of Information Technology at Mayo Clinic. MCLSS contains patient demographics, diagnoses, hospital, laboratory, flowsheet, clinical notes, and pathology data obtained from multiple clinical and hospital source systems within Mayo Clinic at Rochester, Minnesota. Data in MCLSS is accessed via the Data Discovery and Query Builder (DDQB) toolset, consisting of a web-based GUI application and a programmatic API. Investigators, study staff, and data retrieval specialists can utilize DDQB and MCLSS to rapidly and efficiently search millions of patient records. Data found by DDQB can be exported into CSV, TAB delimited, or Microsoft® Excel files for portability. It implements full data authorization and audit logging to ensure data security standards are met.

Note that while DDQB provides graphical user and application programming interfaces for accessing the warehouse database, our goal is to represent the data stored in MCLSS as RDF. In particular, our goal is to create “virtual RDF graphs” which essentially wraps one or more relational databases into a virtual, read-only RDF graph. This will allow us to access the content of large, live, non-RDF databases without having to replicate all the information into RDF. Consequently, for this study, we obtained appropriate approvals from Mayo’s Institutional Review Board (IRB) for accessing patient information in the MCLSS database using programmatic API and JDBC calls (see more details below). Similarly, for accessing the SNP genotype data from MayoGC, we created virtual RDF graphs.

#### RDF virtualization

The RDF virtualization component is based on the freely available Spyder [29] toolkit which acts as mediator in the creation of virtual RDF graphs as well as provides a SPARQL endpoint for querying the graphs. In particular, a declarative language—called the Relational to RDF mapping language (R2RML [30]), an emerging standard under development by the W3C R2RML working group—is used to describe the mappings between the relational schema and RDFS/OWL ontologies to create the virtual RDF graphs. This language generates a mapping file from table structures of the databases in MCLSS and MayoGC, which can then be customized by replacing the auto-generated mappings with concepts and relationships from standardized ontologies. In our case, we replaced the custom ontology mappings with mappings to standardized and community based biomedical ontologies (see below).

#### SPARQL endpoint

The virtual RDF graphs created from MCLSS and MayoGC using the above approach were exposed via a SPARQL endpoint in the Spyder server. This allows software application clients to query the MCLSS and MayoGC RDF graphs using SPARQL. Given that our overarching goal is to integrate the MCLSS and MayoGC RDF graphs, our objective is to execute federated queries across both the SPARQL endpoints. We discuss the details of SPARQL-based federated querying in the subsequent sections.

### Mapping to standardized biomedical terminologies and ontologies

**Figure 2:**
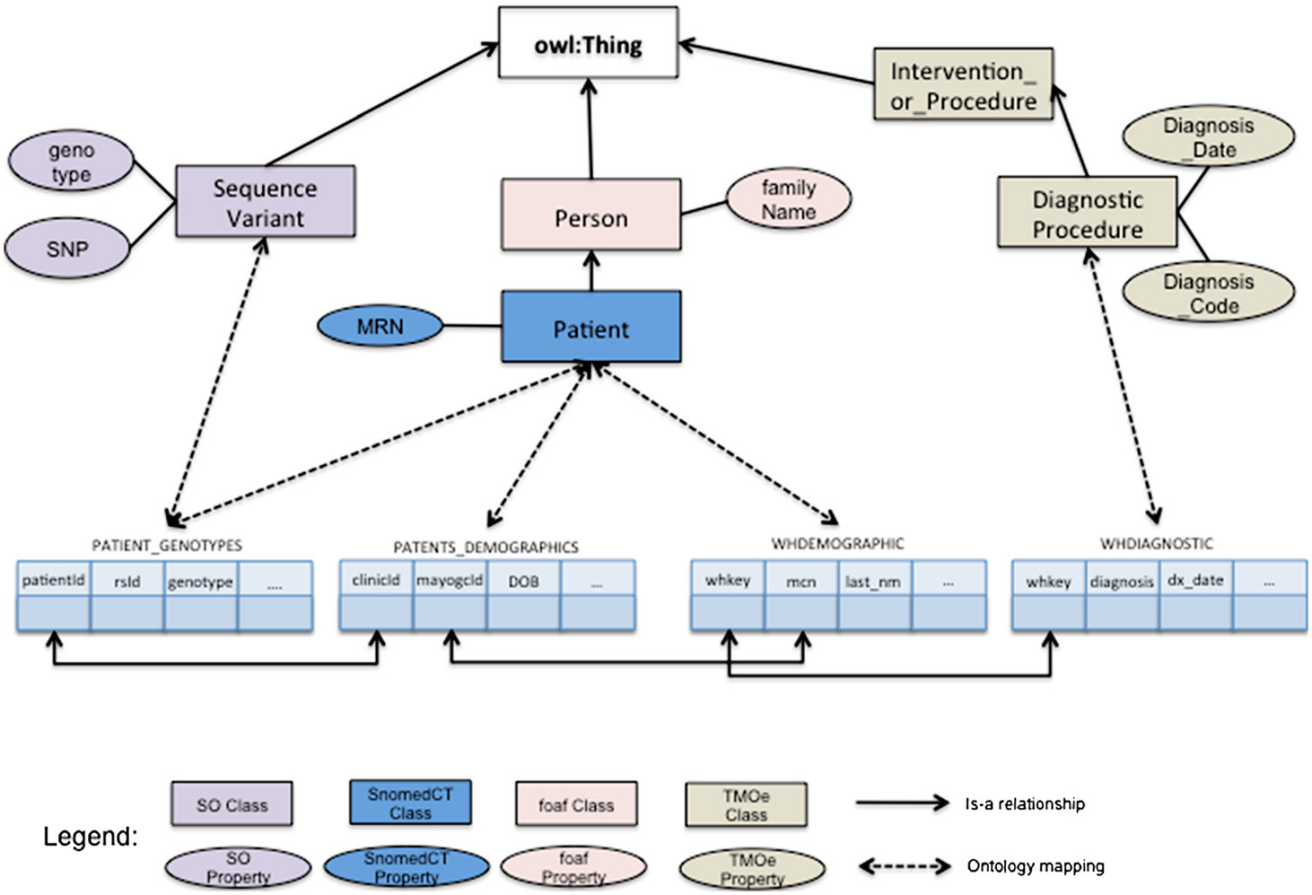
Sample mapping between MCLSS and MayoGC database schemas and existing biomedical ontologies.

**Figure 3:**
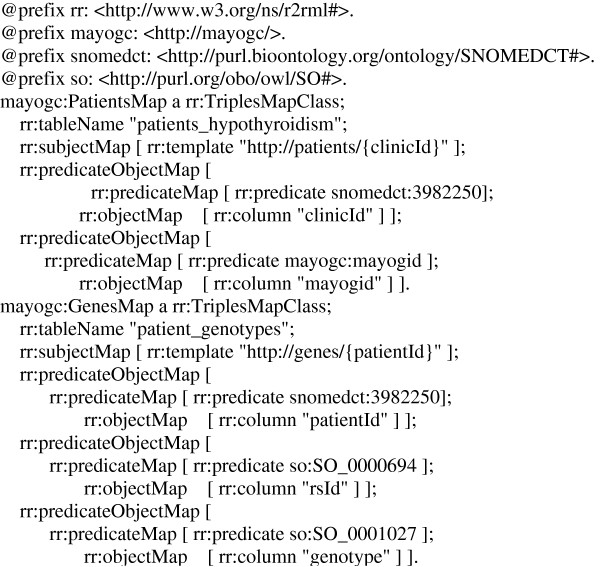
Sample Spyder relational database to RDF mapping file using R2RML.

In its simplest form, any relational schema can be rendered into RDF by converting all primary keys and foreign keys into IRI's, assigning a predicate IRI to each column, and an rdf:type predicate for each row linking it to a RDF class IRI corresponding to the table. Then, a triple with the primary key IRI as subject, the column IRI as predicate and the column's value as object is considered to exist for each column that is neither part of a primary or foreign key. To achieve this goal, we use R2RML and the Spyder toolkit. In particular, for the RDF virtualization and ontology mapping component of our system, we manually create mappings between the MCLSS and MayoGC relational schemas and existing biomedical ontologies, including Translational Medicine Ontology (TMO [31]) and Sequence Ontology (SO [32]), and represent them using R2RML. Of particular relevance to this study is TMO (developed and maintained by a task force of W3C’s Health Care and Life Sciences working group) that aims to model terminological concepts covering several aspects of translational science, including clinical research and drug development. While it provides an overarching structure for representing informational entities from the translational sciences domain, our investigation identified that TMO’s coverage for several core clinical concepts was severely lacking. For example, concepts relevant to a subject’s vital measurements (e.g., body mass index), interventions and procedures, laboratory measurements etc. were not specified as part of the current release of TMO (version 1.0). Consequently, leveraging existing ontologies, namely the Ontology for Biomedical Investigations [33] and Prostate Cancer Ontology [34], we created several new concepts and properties that were subsequently mapped to the NCI Thesaurus [35] and extended the current release of TMO. These extensions can be downloaded from: http://informatics.mayo.edu/LCD. Figures 2 and 3 show a sample of the mappings that were done via R2RML.

### SPARQL-based federated querying for T2DM and Hypothyroidism phenotype-genotype data extraction

**Figure 4:**
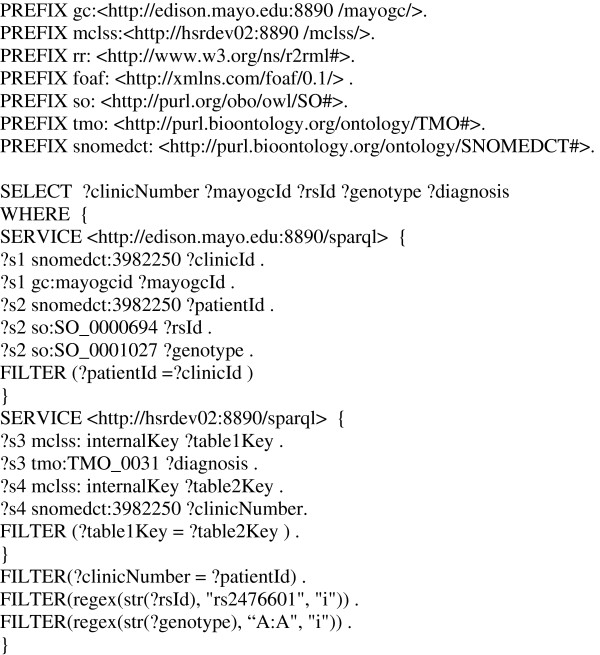
Sample Federated SPARQL query for MCLSS and MayoGC datasets.

As shown in Figure 1, our goal is to federate between two main data sources: MCLSS and MayoGC, where the former is a DB2 database containing patient clinical and demographic data, and the latter is a MySQL database containing genomic information (SNP data) about patients who have volunteered their DNA information to be stored for medical research. Since participation in the Mayo Clinic biobank (and hence, in the MayoGC project) is voluntary, the total number of patients in the MayoGC database is a subset of MCLSS. In its current form, one would have to execute a multiple separate SQL queries across both these databases, for example, to find out the diagnoses for all patients who have a particular SNP genotype, to retrieve an appropriate resultset. Instead, by creating RDF views for MCLSS and MayoGC, we demonstrate how this can be achieved using a single SPARQL query (Figure 4).

In particular, to achieve this goal, two endpoints were created and the SPARQL 1.1 SERVICE keyword was used to access each endpoint. In the first SERVICE stanza, the MayoGC SPARQL endpoint is being queried to provide the MayoGC Identification number for each patient along with their SNP identifiers (*rsID*) and genotypes. Since *clinicId* and *patientId* are primary keys in the relational databases where the information is being stored, the FILTER part of the SERVICE stanza joins the two tables for the query. In the second SERVICE stanza, the MCLSS endpoint is queried to provide the unique Mayo Clinic Identification number for the patient along with their diagnosis data. For the tables in MCLSS, the *internalKey* relationship joins the two tables where their *internalKeys* are equal. The final part of the federated query joins the information retrieved from the two endpoints. The first FILTER statement joins the MayoGC data with the MCLSS data based on the *clinicNumber* and the *patientId*. The final two FILTER statements limit the results for this query to only those patients who, for example, have the SNP “rs2476601” with the genotype “A:A”.

## Results

### Phenome-wide scan for type 2 diabetes mellitus

For evaluating our approach, we first identified the “case” and “control” statuses for all MayoGC subjects by executing the T2DM phenotype criteria defined within the eMERGE consortia [36]. (A “case” status indicates that a subject has been diagnosed with T2DM, whereas a “control” status indicates otherwise). This step was followed by executing the federated SPARQL query illustrated above between the MCLSS and MayoGC endpoints to determine all the subjects having the T2DM SNP genotypes (from Table 2), and retrieving the entire set of ICD-9-CM billing and diagnoses codes for each eligible subject. The reasoning behind using the billing and diagnoses codes was two-fold: (1) these codes are universally used within the U.S. healthcare system, and thereby enables future implementation of our approach at other institutions and multiple EHRs, and (2) the disease, signs and symptoms ICD-9-CM codes can be used as a surrogate for approximating the clinical disease phenotype. However, given that ICD-9-CM was primarily developed for billing and administrative applications and does not necessarily imply a well-defined robust and logical hierarchy for the codes, we used AHRQ’s Clinical Classification Software (CCS [37]) for clustering the billing and diagnoses data into a manageable number of clinically meaningful categories. In particular, CCS classifies over 14,000 diagnoses and 3,900 procedures from ICD-9-CM into 285 and 231 mutually exclusive diagnoses and procedure categories, respectively, that are assigned an unique identifier. This tool is continually updated by AHRQ and the current version used in this study is based on ICD-9-CM codes that are valid from January 1980 till September 2012.

**Figure 5:**
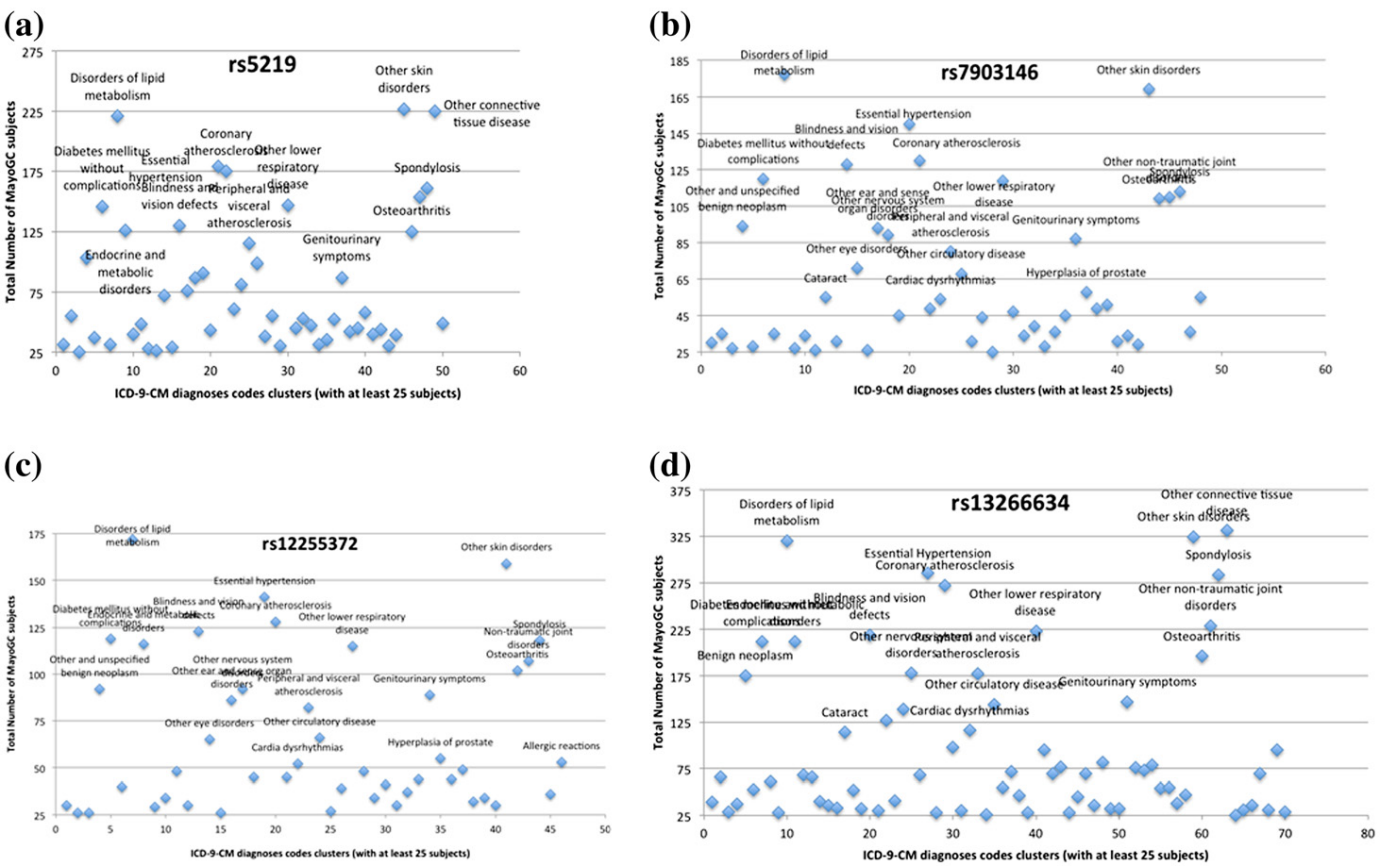
**SNP-disease associations for T2DM SNPs obtained via phenome mining**: (a) SNP rs5219 within the gene KCNJ11; (b) SNP rs7903146 is within the gene TCF7L2; (c) SNP rs12255372 is within the gene TCF7L2; (d) SNP 13266634 is within the gene SLC30A8.

Figure 5 shows the SNP-disease associations for four T2DM SNPs (see Table 2; ICD-9-CM diagnoses codes clusters having less than 25 subjects are not included). There are several observations that are noteworthy. First, for all the four SNPs, we observe a significant association with diabetes and related traits, such as disorders of lipid metabolism. This replicates a finding by Warodomwichit et al [38]. where high (n-6) polyunsaturated fatty acids intakes were associated with atherogenic dyslipidemia in carriers of the minor T allele at rs7903146 SNP in the *TCF7L2* gene and may predispose them to Metobolic Syndrome (MetS), diabetes, and cardiovascular disease. Second, while previous studies have positively associated the SNP rs12255372 (Figure 5(c)) with breast cancer [39] and prostate cancer [40], our findings did not replicate the same association. We believe that this lack of replication is an artifact of the small population size studied in this work. Third, for all the four SNPs, there was a significant association with skin and tissue related diseases (e.g., “Other Skin Disorders”) that included phenotypes such as systemic sclerosis, corns, and seborrhoeic dermatitis. However, further investigation of the literature did not lead to any existing studies where such an association was reported earlier, and thus corroboration of this finding is needed to help rule out a false-positive finding. Finally, since our analysis was done only on 6,307 MayoGC subjects, it is unknown at this time what SNP-disease association patterns will be observed when considering a much larger cohort of subjects from the entire Mayo Clinic Biobank which has approximately 22,000 participants. We discuss all these findings and issues in the Discussion section.

### Phenome-wide scan for hypothyroidism

**Figure 6:**
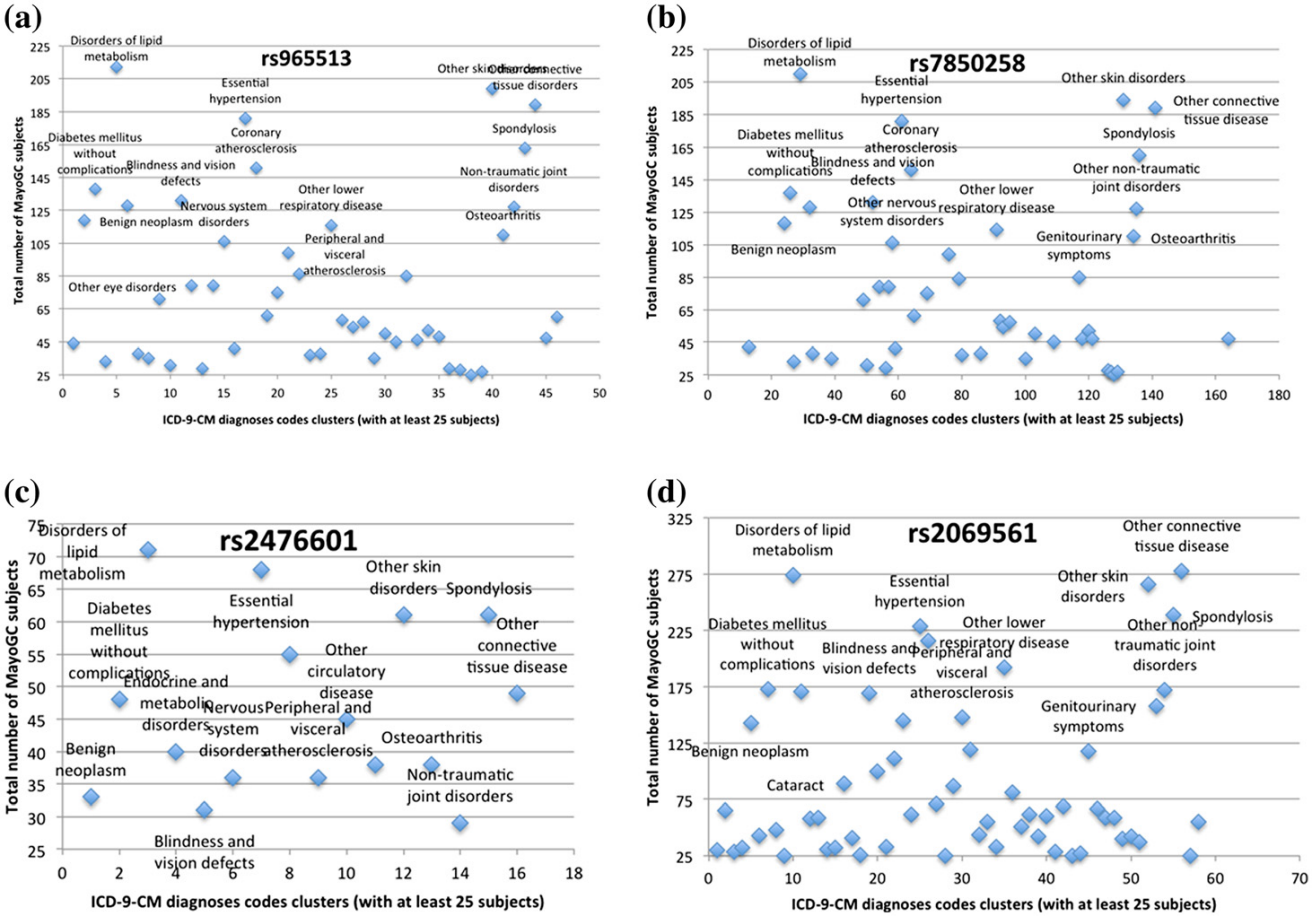
**SNP-disease associations for Hypothyroidism SNPs obtained via phenome mining**: **(a)** SNP rs965513 within the gene FOXE1; **(b)** SNP rs7850258 within the gene FOXE1; **(c)** SNP rs2476601 within the gene PTPN22; **(d)** SNP rs2069561 within the gene TG.

Similar to T2DM, for hypothyroidism, we queried the entire MayoGC cohort of 6,307 subjects for individuals with genotypes for SNPs that have been associated with thyroid disorders (Table 2), and clustered the query results into clinically meaningful categories. Figure 6 shows the SNP-disease associations for four hypothyroidism SNPs (see Table 2; ICD-9-CM diagnoses codes clusters having less than 25 subjects are not included). Similar to the T2DM analysis from above, there are several observations that are noteworthy. First, we observe that compared to the total number of subjects for the SNPs rs965513, rs7850258 and rs2069561, relatively few subjects (n = 136) were identified as having the risk alleles for the SNP rs2476601 (Figure 6 (c)). Second, unlike T2DM, we did not observe a strong association between the four SNPs with any thyroid disorders, including hypothyroidism, hashimoto’s thyroiditis, and congenital hypothyroidism. We hypothesize that this unexpected result is most likely due to the fact that a majority of the subjects in the Phase I of MayoGC cohort have cardiovascular diseases (e.g., n = 1612 with peripheral arterial disease (PAD), n = 1233 with venous thromboembolism (VTE)). Unlike T2DM, while few studies, including one by Biondi and Klein [41], have positively associated hypothyroidism as a risk factor for cardiovascular diseases, compared to other “traditional” risk factors, such as hypertension, the association between thyroid disorders and cardiovascular diseases has not been widely observed. For instance, as evident from the scatter plots in Figure 6 for all the four SNPs, even though we observe a strong association with “essential hypertension”, such an assertion needs further validation and verification. Similarly, the strong associations with skin disorders and related traits require additional investigation. Finally, we see a strong correlation between the hypothyroidism SNPs with blindness and vision defect disorders, such as hypermetropia and amblyopia—a hypothesis that also warrants future studies. As an example, a study by Todd et al [42]. established the correlation between SNP rs2476601 and diabetic retinopathy, which is often regarded as the leading cause of blindness and related vision defects within urban populations.

## Discussion

### Interpretation of results

Research in clinical and translational science demands effective and efficient methods for accessing, integrating, interpreting and analyzing data from multiple, distributed, and often heterogeneous data sources in a unified way. Traditionally, such a process of data collection and analysis is done manually by investigators and researchers, which is not only time consuming and cumbersome, but in many cases, also error prone. The emerging Semantic Web tools and technologies allow exposing different modalities of data, including clinic, research, and scientific, as structured RDF that can be queried uniformly via SPARQL. Not only does this provide the capabilities for interlinking and federated querying of diverse data resources, but also enables fusion of private/local and public data in very powerful ways for secondary uses.

The overarching goal of this study is to investigate Semantic Web technologies for federated data integration and querying using real clinical and genetic data from Mayo’s EHR and biobank. Using open-source tooling and software, we developed a proof-of-concept system that allows representing patient clinical and genotype data stored in Mayo’s enterprise warehouse system (MCLSS) and the MayoGC databases, respectively, as RDF, and exposing it via SPARQL endpoints for accessing and querying. We leveraged existing ontologies, such as the Translational Medicine Ontology, Ontology for Biomedical Investigations and Sequence Ontology for mapping the MCLSS and MayoGC database schemas to standardized semantic concepts and relationships. Our use case for doing a phenome-wide association scan for two chronic diseases, namely T2DM and hypothyroidism, demonstrated the applicability of using such an approach for flexibly interlinking and querying multiple heterogeneous data sources in a robust and semantically unambiguous manner. We hypothesize that further development of such a system can immensely facilitate, and potentially accelerate scientific findings in clinical and translational research, including personalized medicine and systems biology.

The ultimate challenge for any PheWAS study is data interpretation. While discovery of new genotype-phenotype associations in PheWAS is important, many of the findings may reflect inter-relationships existing among the phenotypes, sub-phenotypes and endo-phenotypes. As observed in the study by Pendergrass et al [9]., the “novel” results may exemplify pleitropy. For instance, as discussed earlier, all the four SNPs that have been previously associated with diabetes and related traits in Figure 5, also demonstrate a significant association with skin and tissue disorders, thereby indicating the impact of genetic variation on the genes to both phenotypes. Since PheWAS is meant to generate hypothesese, and hence by nature is exploratory, further investigation within large cohort sizes is required to validate such findings.

### Limitations

There are several limitations in the proof-of-concept system developed as part of this study. First, while we demonstrated the applicability of the system via two simple use cases for T2DM and hypothyroidism, a more robust and rigorous evaluation along several dimensions (e.g., performance, query response, precision and recall of query results etc.) is required before it can be deployed within an enterprise environment. Note that since our use cases are based on federated querying of multiple SPARQL endpoints, the system performance and query responses are dependent on the behavior of the endpoints (e.g., the endpoints may experience latency, denial of service). Nevertheless, we plan to perform a thorough system evaluation after the integration of additional MCLSS sources (e.g., laboratory, clinical and pathology reports) that contain large amounts of patient data. Second, we experimented with the recently published Translational Medicine Ontology (TMO) in this study for mapping between MCLSS database schemas to standardized concepts and relationships. While TMO classes are mapped to more than 60 different standardized ontologies, including SNOMED CT and NCI Thesaurus, the scope and breadth of the current TMO release (Version 1.0) is significantly narrow for our purpose. Consequently, along with the creation new classes and relationships, we augmented TMO with Prostate Cancer Ontology and the Ontology for Biomedical Investigation. Since these extensions are not part of the official TMO release, our goal is to work closely with the TMO curators for content enhancement in future releases. Third, formulating the complex SPARQL queries using existing SPARQL editors is cumbersome and error prone. Our current implementation lacks a more intuitive and user-friendly interface that can assist a “non-Semantic Web savvy” user in the query building process. We plan to address this issue within the timeframe of the project by investigating multiple open-source graphical SPARQL editors. Finally, while in this study we only considered T2DM and hypothyoroidism as our use cases, in the future we plan to conduct a large-scale PheWAS with the entire Mayo Clinic Biobank population, which currently has more than 22,000 subjects enrolled as of June 2012.

### Future work

In addition to addressing the aforementioned limitations, there are several activities that we plan to pursue in the future. Firstly, in this study, we performed simple mappings between the MCLSS and MayoGC database schemas to classes and relationships in several biomedical ontologies including TMO and Sequence Ontology. A more rigorous approach will be to investigate reference information models, such as clinical archetypes [43], that provide a mechanism to express data structures in a shared and interoperable way. Secondly, we will investigate existing Semantic Web querying visualization platforms such as SPARQLMotion [44] and TripleMap [45] that provide more intuitive and user-interactive interfaces for SPARQL query formulation and execution. We also plan to provide API-based access to software clients, and for this, we will experiment with the recently released open-source Elda [46] Linked Data API. Finally, we will investigate approaches for distributed and federated inferencing over RDF data. Recent studies [47] have demonstrated that even simple subsumption inferences require significant computing power when reasoning over massive RDF datasets. Since access to extremely high-performance computers is not readily available en masse, we will investigate distributed storage and indexing techniques using Apache Hadoop [48].

## Conclusions

This study demonstrates how Semantic Web technologies can be applied in conjunction with clinical data stored in EHRs to accurately identify subjects with specific diseases and phenotypes, and perform a PheWAS by integrating and analyzing the genotype data with a range of phenotypes. Such an approach has the potential to immensely facilitate the tedious, cumbersome and error prone manual integration and analysis of data for clinical and translational research, including genomics studies and clinical trials.

## Competing interests

The authors declare that they do not have any competing interests.

## Authors’ contributions

JP and RCK designed and implemented the system. JP and RCK wrote the manuscript. SJB and CGC provided assistance in the study design and analysis of the results. All authors read and approved the final manuscript.

## Availability of supporting data

Information and details about the software and relevant third-party applications described in this manuscript is available from http://informatics.mayo.edu/LCD.
